# Green synthesis of biocompatible Fe_3_O_4_ magnetic nanoparticles using *Citrus Sinensis* peels extract for their biological activities and magnetic-hyperthermia applications

**DOI:** 10.1038/s41598-023-46287-6

**Published:** 2023-11-03

**Authors:** Bahig A. Eldeeb, Walaa M. Abd El-Raheem, Shehab Elbeltagi

**Affiliations:** 1 Department of Botany and Microbiology, Faculty of Science, Sohag University, Sohag, Egypt; 2 Department of Physics-Biophysics, Faculty of Science, New Valley University, El-Kharga, 72511 New Valley Egypt

**Keywords:** Nanoscale biophysics, Biophysics, Nanoscience and technology

## Abstract

Green synthesis of nanoparticles (NPs) is eco-friendly, biocompatible, cost-effective, and highly stable. In the present study*, Citrus sinensis* peel extract was utilized to the fabrication of superparamagnetic iron oxide nanoparticles (SPIONs). The fabricated SPIONs were first characterized using UV–Visible spectroscopy, Fourier transform infrared (FTIR) spectroscopy, X-ray diffraction (XRD), Transmission electron microscopy (TEM), and vibrating sample magnetometer (VSM). The UV–Vis spectra analysis displayed a peak at 259 nm due to the surface plasmon resonance. The FTIR spectrum showed bands at 3306 cm^−1^, and 1616 cm^−1^ revealed the protein’s involvement in the development and capping of NPs. TEM analysis indicated that green synthesized SPIONs were spherical in shape with particle size of 20–24 nm. Magnetization measurements indicate that the synthesized SPIONs exhibited superparamagnetic behavior at room temperature. The antimicrobial activity, minimum inhibitory concentration (MIC), antioxidant potential, anti-inflammatory effect, and catalytic degradation of methylene blue by SPIONs were investigated in this study. Results demonstrated that SPIONs had variable antimicrobial effect against different pathogenic multi-drug resistant bacteria. At the highest concentration (400 μg/mL), SPIONs showed inhibition zones (14.7–37.3 mm) against all the target isolates. Furthermore, the MIC of synthesized SPIONs against *Staphylococcus aureus, Streptococcus mutans*, *Bacillus subtilis*, *Escherichia coli*, *Klebsiella pneumonia*, and *Candida albicans* were 3, 6.5, 6.5, 12.5, 50, 25 μg/mL, respectively. SPIONs exhibited strong antioxidant, anti-inflammatory, and catalytic dye degradation activities. Interestingly, Fe_3_O_4_ SPIONs shows optimum magnetic hyperthermia (MHT) techniques under an alternating magnetic field (AMF) measured in specific absorption rate (SAR) of 164, 230, and 286 W/g at concentrations 1, 5, and 10 mg/mL, respectively. Additionally, these newly fabricated SPIONs virtually achieve significant execution under the AMF in fluid MHT and are suitable for biomedical applications.

## Introduction

Nowadays, the effects of NPs on various microorganisms have attracted significant attention from the scientific community as an alternative to antibiotics^1^. The World Health Organization (WHO) finds it particularly concerning that multi- and pan-resistant bacteria are rapidly spreading worldwide and causing diseases that are not treatable with the current antimicrobial medications such antibiotics, antivirals, antifungals, etc.^2^. Studies using diverse plant components, including leaf, flowers, roots, stems, seeds, and peels extracts, to biosynthesize NPs have drawn attention to use plant extracts as platforms for MNP synthesis. Various biological, therapeutic, and pharmacological fields have utilized plants^3–7^. SPIONs can be made in multiple ways, including thermal breakdown, co-precipitation, and green biosynthesis, the last well-established technique that can yield significant SPIONs^8–12^. The HT temperature and Fe_3_O_4_ NPs' characteristics in MHT can be controlled by the AMF strengths^13^. HT is classified into three types: regional HT, local HT, and whole-body HT. The ferrofluid samples’ specific absorption rate (SAR) is measured by exposing them to an external alternating magnetic field (AMF)^14^. MHT cancer treatment, biosensors, and drug delivery are some potential therapeutic applications for SPIONs with nano-sizes less than 50 nm^15–17^. The biocompatibility SPIONs are frequently utilized in wastewater treatment of several organic dyes as a ferrofluid, metal adsorbent, antibacterial agent, and antifungal reagent^18–24^, that reduce the amount of dissolved oxygen in water and threaten the aquatic ecology^25^. The effects of SPIONs on bacteria and pathogens have received very little research, even though MNPs doped with several metals have antibacterial properties^26,27^. Due to their chemical biostability, excellent biocompatibility in terms of body clearance, safety, simplicity of surface coating, and superparamagnetic performance, SPIONs have attracted the most interest in HT research^28–30^.

Koli et al.^31^ reported that a facile and cost-effective green synthesis of Fe_3_O_4_ biocompatible MNPs with particle size of 13 nm, its practical application in MHT, and impact of various physical parameters on SAR of MNPs. Mostafa et al.^13^ the green synthesis of Fe_3_O_4_ nanofluids by using the crude extract of *G. mangostana fruit peel* and potentially used in HT, since they mostly caused the heating increase at the secure HT range (42–47 °C) and the acceptable SAR values under AMF. SPIONs are still considered a more optimized alternative for HT treatment, and current research focuses on adjusting their size, stage of aggregation, and crystalline phase structure to enhance their thermal properties^32^. This technique generates thermal energy, which is subsequently used to manufacture HT and destroy cancer cells by raising the target tissues' temperature over 43 °C, which is suitable for handling HT^33,34^.

In current research, the Fe_3_O_4_ SPIONs were fabricated by green synthesis using an eco-friendly approach. For the biological activities, SPIONs were also applied as antibacterial agents against harmful bacterial strains such as *Escherichia coli*, *Streptococcus mutans*, *Staphylococcus aureus*, *Bacillus subtilis*, *Klebsiella pneumonia,* and as antifungal agents against *Candida albicans*. Using a DPPH assay, the antioxidant activity of SPIONs was confirmed. Additionally, this work demonstrated that SPIONs are an effective strategy to enhance their thermal efficiency; SAR values of SPIONs were determined and examined for HT.

## Materials and methods

### Ethical approval

The samples were collected for the present study and mediated by the regulation of the New Valley Research Ethics Committee (NVREC), Faculty of Science, Physics Dep., New Valley University, Egypt, and the work was approved under license Protocol Number: 06–3-6–2023-1.

### Preparation of *Citrus sinensis* peel extract

**Figure 1:**
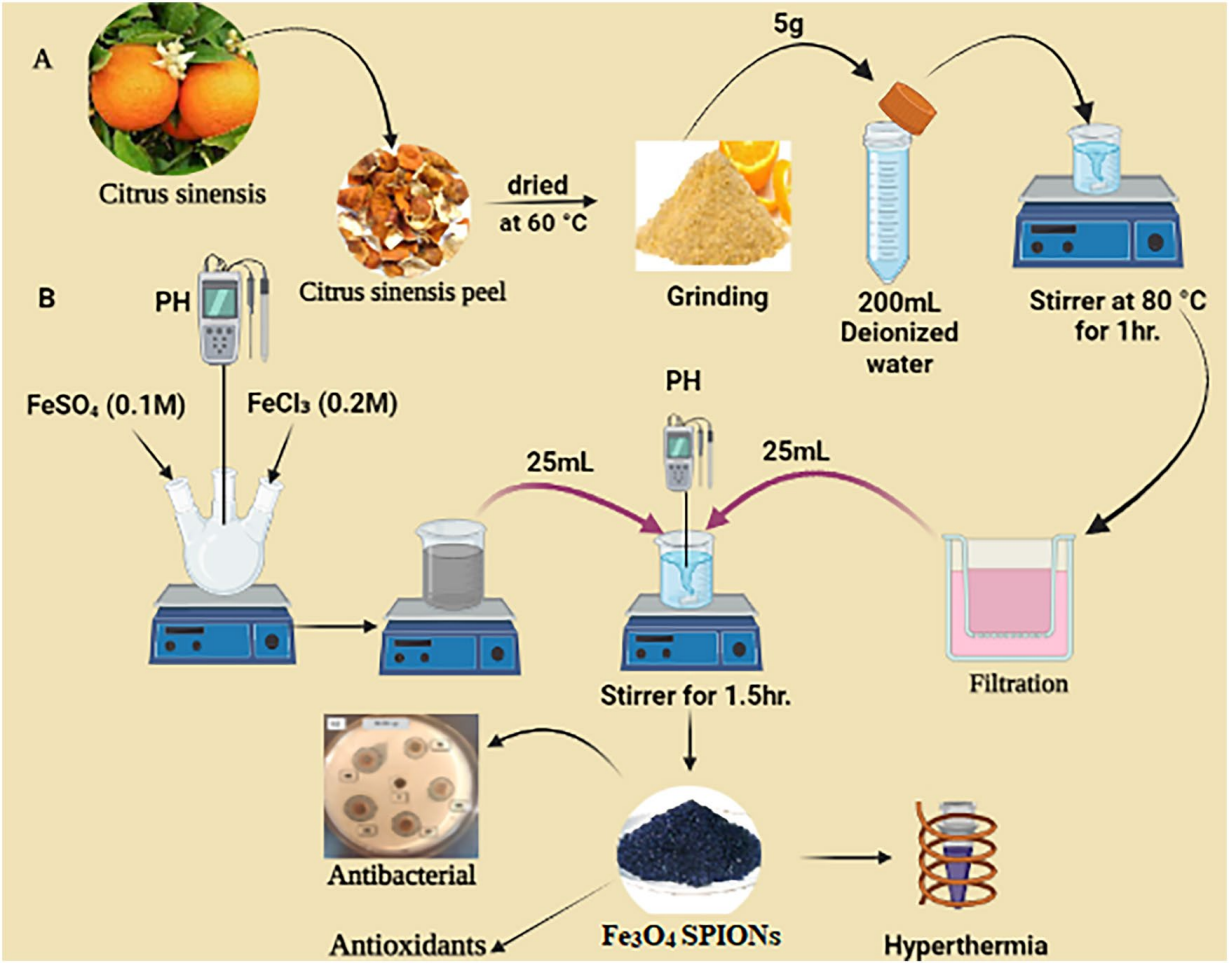
Schematics of the biosynthesis of SPIONs from *Citrus sinensis* peel extract and their applications, (schematic created by Biorender.com).

The peels were cleaned, dried for 72 h at 60 °C, ground into a fine powder, and mixed with double-distilled water (5 g in 200 mL) to create an extract (Fig. 1A). The extract was filtered twice via Whatman No. 1 filter paper, and stored at 4 °C in a tight glass bottle for further synthesis of Fe_3_O_4_ NPs^35^.

### Synthesis of SPIONs

The synthesis of SPIONs were carried out by using ferric chloride hexahydrate (FeCl_3_·6H_2_O) (0.2 M) and ferrous sulfate heptahydrate [FeSO_4_·7H_2_O] (0.1 M) in a 2:1 mol ratio (Fig. 1B). A 25-mL solution was added to 25-mL of peel extract and stirred with magnetic stirrer at room temperature for 30 min, the pH of the mixed solution was adjusted to 11–12 using 0.1 M NaOH, and a dark black precipitate formed immediately^36^. The formation of SPIONs was identified by the starting mixture color altering from yellow to black. After that, the black material was allowed to dry at room temperature after being washed three times with double-distilled water and twice with ethanol.

### Characterizations of SPIONs

#### UV–Vis spectrophotometry

The production of the SPIONs was investigated by scanning SPIONs suspension using UV–Vis spectrophotometer (JASCO V-770 Spectrophotometer), with spectra ranging from 200 to 800 nm. Distilled water was used as blank.

#### FT-IR analysis

FTIR measurement was performed to confirm the functional groups of biosynthesized SPIONs by Fourier transform infrared spectrometer (Bruker, Germany, Alpha-P). The SPIONs suspension was dropped on the sample holder. The spectrum absorbance of samples was recorded in the range of 4000–400 cm^−1^ at a resolution of 4 cm^-1^.

#### XRD

XRD is the most commonly used method for analyzing the chemical component and figuring out the size and crystal shape of SPIONs. Revealing of the SPIONs’ characteristics by XRD goes through specific steps starting from casting a beam of X-rays on the crystals, then the fallen beam dispersed via the atoms, which leads to the emergence of diffraction patterns followed by the interference of beams with each other^37^. The Scherrer formula can be utilized for determining the domain size of the crystal material (1)^38^:

1 $$\mathrm{D}=\frac{\mathrm{k\lambda }}{\beta cos\theta }$$

where β is the corrected full width at half maximum (FWHM) in radians and k = 0.90 is shape constant, k is the target wave length k = 1.54060 Å.

#### TEM

TEM is an effective imaging technique that used to evaluate the morphological size and properties of SPIONs by TEM JEOL Model JSM-5400 LV (Joel, Tokyo, Japan). TEM interacts with the specimen (SPIONs) via electrons beam to pattern the image on the photographic plane. Creating a high-resolution picture of the atomic space of SPIONs is an amazing application of TEM. The catalyst powder dispersed in ethanol using ultrasonic radiation for 20 min and a drop of that suspension was placed onto the carbon-coated grids. The degree of magnification of TEM images was the same for all the different investigated catalysts.

#### Magnetic hyperthermia

**Figure 2:**
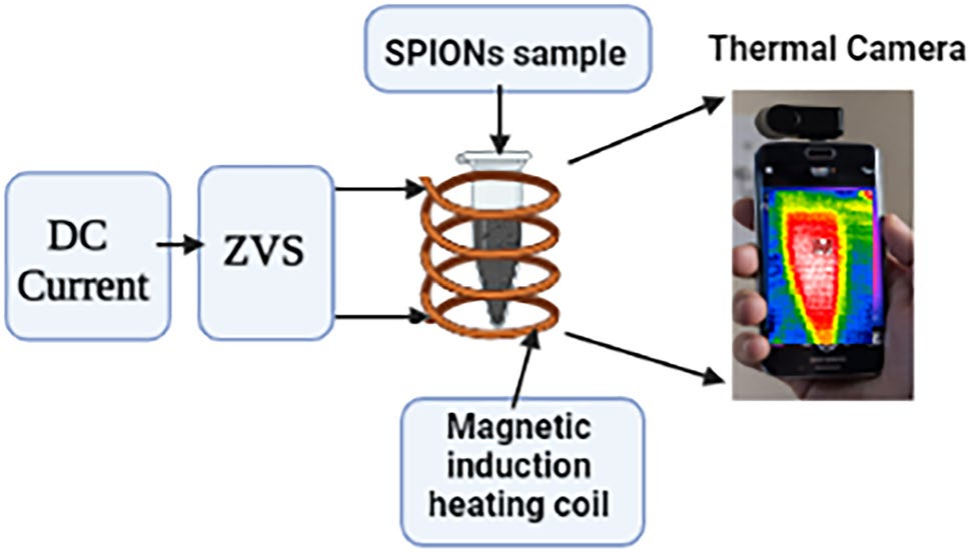
Magnetic induction heating set-up.

The heating ability of magnetic fluid is called SAR. The thermal efficiencies of SPIONs in HT equipment were evaluated using our system^39^ (Fig. 2). The system converts the DC into AMF is used in a magnetic induction heating setup, and a sample container that is insulated is put inside a copper solenoid coil^40^. The water-cooled copper tube heating coil had a 3 cm radius and a 7-turn radius and was composed of copper tubes. The magnetic HT tests were conducted using 14.6A DC generators, a variable magnetic field strength of 6.3 kA/m, and a fixed frequency of 290 kHz. The sample temperature was maintained by lowering the magnetic field intensity once it had been reached. A thermal camera captured the samples' heating every 1 min, and the SAR (W/g) for each sample was determined from the initial slope (dT/dt) of the temperature fluctuation by time^41^.

#### Antimicrobial efficiency

The antimicrobial potency was identified via well diffusion assay against the multi-drug resistant pathogens (*Staphylococcus aureus*, *Streptococcus mutans, Bacillus subtilis, Escherichia coli, Klebsiella pneumonia* and also *Candida albicans*). Pure cultures of the test specimens were sub-cultured in nutrient broth for bacteria and Sabouraud dextrose broth for *Candida* then each strain was uniformly spread on sterilized petri plates with Muller-Hinton agar. A circular well of 7 mm diameter was made in plates using a sterile cork-borer. The wells were loaded with (50 µL) of different concentrations of the biosynthesized SPIONs (50, 100, 200, 300, and 400 μg/mL) and incubated for 24 h at 37 °C for bacteria and for 2–3 days at 28 °C for *Candida*. As a control, DMSO was used. The zone of inhibition was measured by a scale to the nearest mm including disc diameter^42^.

#### Determination of the minimum inhibitory concentration (MIC)

Minimum inhibitory concentration (MIC) for SPIONs was assayed using microtiter plate technique. A serial dilution (50, 25, 12.5, 6.5, 3, and 1 μg/mL) of SPIONs were tested against the target pathogens. From each dilute, 0.1 mL was added to 5 mL NBPG medium; Nutrient broth containing 0.05% phenol red and added with 10% glucose. One hundred microliters of each concentration were added to a well (96-wells micro plate) containing 95 μL of NBPG and 5 μL of the tested microbial suspension containing 10^6^ CFU/mL. The negative control well contained the same mixture without adding SPIONs. The plates were covered and incubated for 24 h at 37 °C for bacteria and at 28 °C for 4 days for *Candida* isolate, the assay was repeated twice. Microbial growth was identified by noting the color change in the wells (red when there is no growth and yellow when there is growth). The lowest concentration of the SPIONs showing no color change was considered as the MIC.

#### Antioxidant activity

The antioxidant activity of SPIONs was identified by a 2,2-diphenyl picrylhydrazyl (DPPH) scavenging assay^43^. First, the different concentrations of SPIONs (50, 100, 150, 200, 250, and 300 µg/mL) were dissolved in distilled water. Then, 1 mL of SPIONs was mixed with 1 mL of 0.1 mM DPPH (freshly prepared) in each test tubes. The solution was combined well and incubated in the dark room for 30 min. After incubation, the absorbance of the solution was recorded at 517 nm using UV-spectrophotometer (JASCO V-770 Spectrophotometer). DPPH was used as blank and ascorbic acid as standard. % DPPH radical scavenging activity determined by the formula (2)^44^:

2 $$\frac{\mathrm{A}0-\mathrm{As }}{\mathrm{A}0}\times 100$$

where A_0_ is the absorbance value of the control and A_s_ is the absorbance value of the sample.

#### In vitro anti-inflammatory

The bovine serum albumin denaturation assay was performed in accordance with the method followed by Sakat^45^. A reaction solution (0.5 mL) was made by adding bovine serum albumin (0.45 mL) and different concentrations (50–300 μg/mL) of the SPIONs (0.05 mL). Phosphate buffer (2.5 mL; pH 6.3) was combined with the reaction solution, and the contents of test tubes were incubated at 40 °C for 25 min then heated for 5 min at 70 °C. After cooling at room temperature, the A_600_ of the solution was determined. Diclofenac sodium and phosphate buffer solution were used as positive and negative controls, respectively. The percent inhibition rates are calculated using the following Eq. (3)^46^:

3 $$\mathrm{Inhibition\,\%}=\frac{ {\mathrm{Abs}}_{\mathrm{Control}} - {\mathrm{Abs}}_{\mathrm{treated}} }{{\mathrm{Abs}}_{\mathrm{Control}}}\times 100$$

#### Catalytic reduction of methylene blue

The catalytic efficacy of biosynthesized SPIONs was investigated for the degradation of methylene blue dye, with the application of NaBH_4_. For this, 2 mM aqueous dye and 0.03 M of freshly prepared NaBH_4_ solutions were added to biosynthesized SPIONs, and the change in the concentration of methylene blue with time was monitored by UV Visible spectrophotometer.

## Results

**Figure 3:**
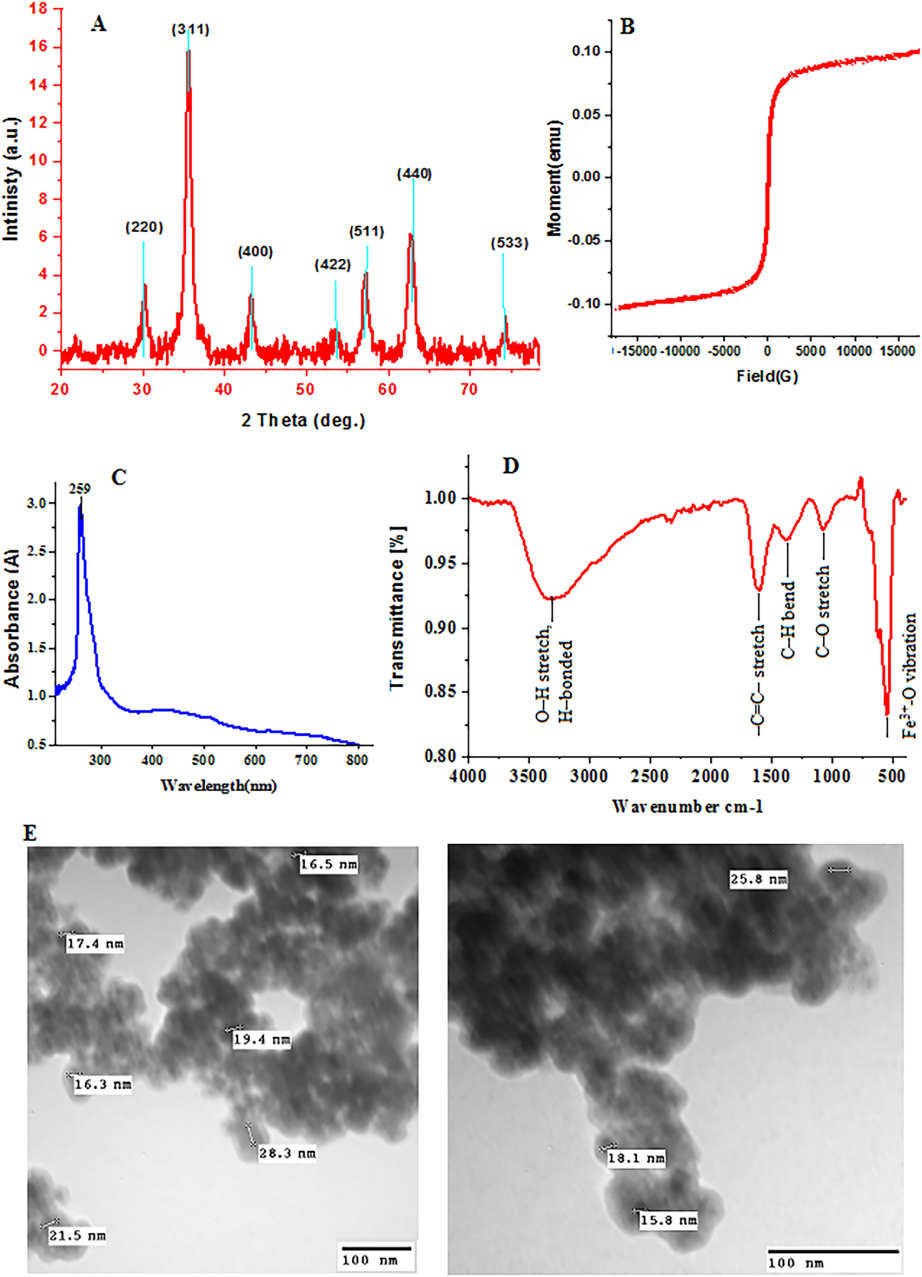
(**A**) X-ray diffraction, (**B**) VSM, (**C**) UV–Vis analysis spectrum, (**D**) FT-IR spectrum, and (**E**) TEM.

The XRD of SPIONs crystal structure was investigated (Fig. 3A). The generated SPIONs produced diffraction peaks corresponding to the Face-Centered Cubic (FCC) phase of metallic iron, resulting in a highly crystalline structure. The sample's XRD profiles exhibit a wide range of peaks, with the maximum intensity of Bragg reflection occurring at 2θ = 36°. It shows diffraction peaks at 2*θ* values of 18.9°, 35.4°, 28.6°, 43.1°, 53.7°, 57.2°, and 62.8° which are corresponding to (111), (311), (220), (400), (422), (511) and (440) crystal planes of cubic phase of SPIONs with a space group of Fd3m (JCPDS card No. 74-2081)^47^. These peaks are consistent with the standard pattern (JCPDS No. 19-629), which reported that the magnetite was pure iron oxide with a cubic inverse spinal structure^48^, which suggests that the SPIONs are resistant to oxidation by oxygen in the air due to their capping with biomolecules from *Citrus sinensis* peel extract.

Figure 3B shows the VSM magnetization curves of SPIONs; the saturation magnetization (M_*S*_) frequency of magnetically functionalized SPIONs has been distinguished using a VSM. Inter- and intra-particle interactions have an impact on the magnetic property. Several elements influence these interactions, including the SPION shell thickness and core size. The magnetization value of bulk SPIONs is approximately 102emu as well.

Visualization of the biosynthesis of superparamagnetic SPIONs using the peel extract of *Citrus sinensis* was safe, simple and cost-effective method. Synthesis of SPIONs was observed by alter in color of the mixture from yellowish to brown then black. UV–Visible spectrum of SPIONs showed the characteristic peak at 259 nm, as demonstrated in (Fig. 3C) which confirmed the presence of iron oxide nanoparticles^49^.

FT-IR spectra were also used to determine which functional groups were present in the biosynthesized SPIONs (Fig. 3D). The spectrum of SPIONs displayed the absorption peaks at: 3306 cm^−1^ (O–H stretching vibration), 1616 cm^−1^ (the stretching vibration of the N–H bend the amide 1 group), 1373 cm^−1^ (C–N stretching of aromatic amines), 1070 cm^−1^ (stretching vibration of –C–O), and 558 cm^−1^ (Fe^3+^-O stretching vibration). TEM micrographs of the particle size and distribution histograms of the SPIONs are shown in (Fig. 3E). The image reveals uniform distribution with a mean value of 20 nm closely match the restricted size distribution of the SPIONs.

### Biological activities of SPIONs

#### Determination of antimicrobial activity

**Figure 4:**
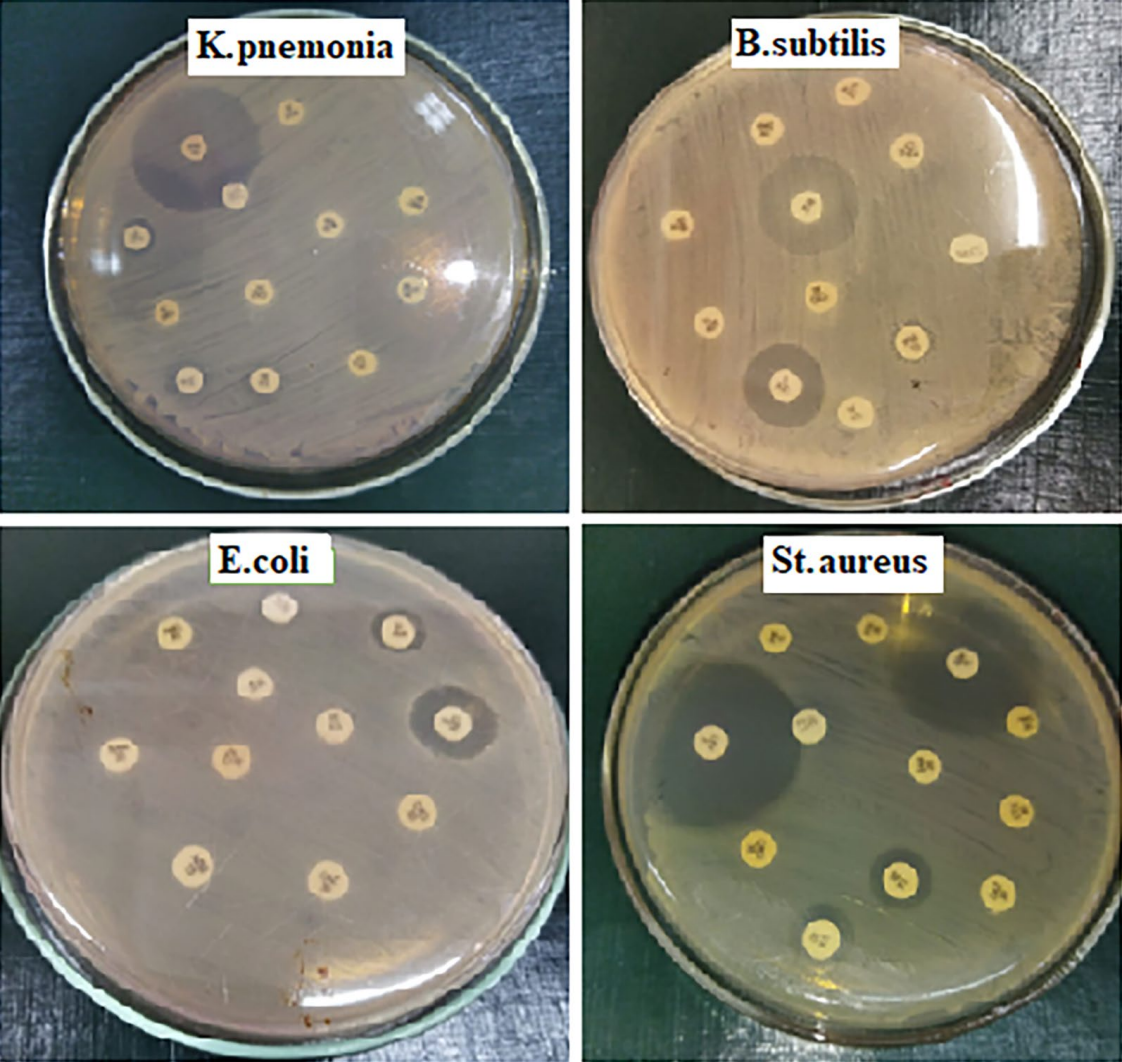
Antibiotic profile of the tested pathogens.

**Figure 5:**
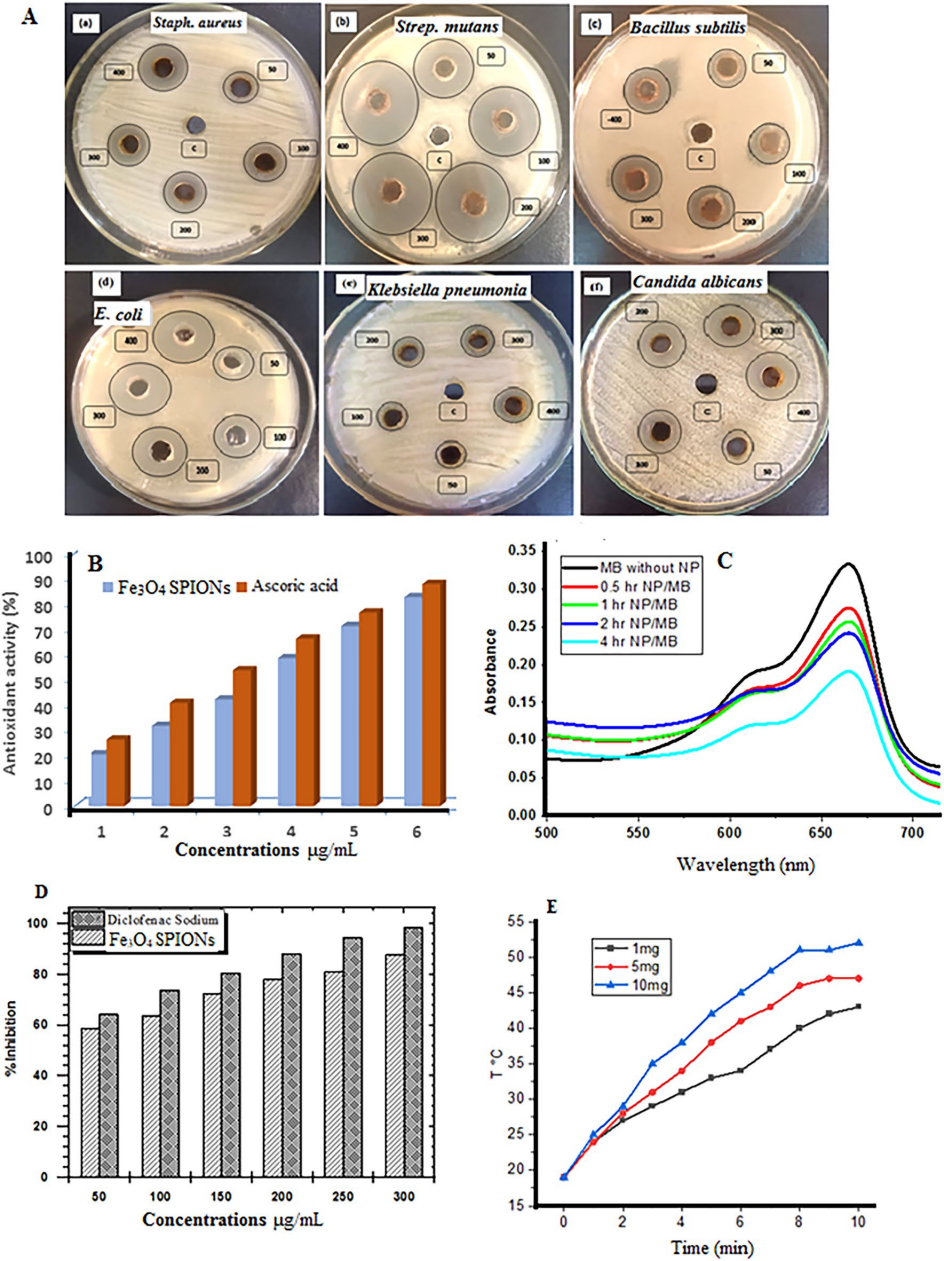
(**A**) Antimicrobial activity, (**B**) The antioxidant activity, (**C**) Catalytic dye degradation, (**D**) Inhibition% Vs. concentration of protein denaturation, and (**E**) Curves of temperature versus time.

In response to microbial multidrug resistance problems (Fig. 4), NPs from natural sources could be possible alternatives to antibiotics. The fabricated SPIONs were measured for antimicrobial properties against the clinical bacterial isolates which displayed resistance to most of the standard antibiotics. The antimicrobial potency of SPIONs was evaluated by the agar-well diffusion method. The diameter of inhibition zone produced by five different concentrations (50, 100, 200, 300, and 400 µg/mL) of the SPIONs are given in Table S1and (Fig. 5A).

#### Minimum inhibitory concentration (MIC)

MIC of the SPIONs was estimated using microtiter plate technique. The lowest concentration of the antimicrobial material, which did not show any visible growth, was considered as the MIC^50,51^. The results were illustrated in Table S1. The MIC of green synthesized SPIONs against *Staphylococcus aureus, Streptococcus mutans, Bacillus subtilis, Escherichia coli, Klebsiella pneumonia* and *Candida albicans* were 3, 6.5, 6.5, 12.5, 50, 25 μg/mL, respectively, since the color of the wells changed from red to yellow at these values indicating the presence of bacterial growth. The wells of negative control were yellow in color due to an absence of SPIONs which inhibit the growth of the bacterial isolates.

#### Antioxidant activity analysis

Reactive oxygen species (ROS), which cause oxidative damage, are prevented by antioxidants from harming the cells. The antioxidant activity of SPIONs was validated using DPPH assay. When iron oxide is present, the color of the DPPH mixture progressively changes from deep violet to pale yellow, allowing for the visual evaluation of the NPs' antioxidant activity. Figure 5B displayed the antioxidant activity of various concentrations of SPIONs (50–300 μg/mL). At the highest concentration, SPIONs illustrated the potent DPPH radical scavenging of 82.6%, while standard ascorbic acid showed 87.8% antioxidant activity.

*The catalytic activity of the biosynthesized SPIONs* was investigated by using the reduction of MB dye in the presence of NaBH_4_ at room temperature. The high absorbance of the MB is located at the wavelength of 666 nm. The catalytic reactions were monitored by using UV–Vis spectroscopy. After adding NaBH_4_ aqueous solution and SPIONs as a catalyst, dye absorbance decreased with time (Fig. 5C). The decreasing process was not detected in the absence of catalyst, even in the existence of excess amounts of NaBH_4_ so the results showed that the SPIONs are more efficient catalyst with respect to reaction time.

#### Evaluation of anti-inflammatory activity of SPIONs by albumin denaturation assay

At all of the measured concentrations of the SPIONs (50–300 µg/mL), there was significant resistance to albumin denaturation in a concentration-dependent manner. At the highest measured concentration (300 µg/mL), the maximum percentage of inhibition was 88%. Furthermore, at a concentration of 300 µg/mL, Diclofenac, which was utilized as a control medication, showed a maximal inhibition of 98% (Fig. 5D).

#### Magnetic-hyperthermia performance and SAR values

The thermal efficiency of SPIONs, which is measured as the SAR with (W/g) units, which is the thermal power generated per mg of magnetite NPs when they are exposed to AMF, determines whether or not they are suitable for HT treatment (Fig. 5E). In our work, we used Eq. (4)^52^ to determine SAR values:

4 $$SAR={C}_{e }\left(\frac{{m}_{s}}{{m}_{m}}\right) \frac{dT}{dt}$$

where m_m_ is the mass of magnetic material in suspension, C_e_ = (4.186 J/g °C) is the specific heat suspension capacity, m_s_ is suspension mass, and dT/dt is initial slope of temperature–time plot in the linear region. The SAR values for SPIONs were 164, 230, and 286W/g for different concentrations of 1, 5, and 10 mg, respectively Table S2, reported literature on SPIONs and SAR values^13,53–55^.

## Discussion

*The XRD results* of the SPIONs confirm that the magnetic powders of black color are Fe_3_O_4_ in nature and have excellent crystallinity with a high purity structure. The observed peak amplitude corresponds to the small particle size^56^, and the density theoretical was computed from Eq. (5)^45,57^:

5 $${D}_{x}=\frac{ZM}{NV}$$

where Z is the molecules per unit cell number, N is Avogadro’s number, V is the unit cell volume (V = a^3^) in a cubic symmetry case, and M is the molecular weight. From Scherrer's Eq. (1), the nanocrystallite diameter (D) is essential because all diffraction profiles exhibit broad profile distribution as a result of X-ray scattering from small nanocrystals. From the shape parameters of all peaks, nanocrystallite size was calculated from (6)^40,58^.

6 $$\mathrm{SSA}=\frac{6000}{\mathrm{D}\cdot {\mathrm{D}}_{\mathrm{x}}}$$

where D is the nanocrystallite size, and D_x_ is the theoretical density, so the nanocrystalline size was found to be 23.4 nm.

According to VSM measurements, the M_*S*_ value of magnetic SPIONs nanocomposite is 102 emu, which relates to non-magnetic shells excessing in the nanocomposite^59,60^. The decreasing nanocrystallite size from XRD data, growing lattice strain, and the presence of Fe^2+^ ions in the system^61,62^, where increasing anisotropy is a constant value, may support the increasing coercivity (H_c_) value of 286emu. The UV–Visible spectra for SPIONs is appropriate surface plasmon resonance (SPR) by greater band strength at 259 nm was used to demonstrate how *Citrus sinensis* peel extract reduces the SPIONs. SPR phenomena excitation can be used to explain why the precursor solution color changed during the formation process from yellow to black^23,63,64^.

The FTIR indicated that the bonds C-O stretching vibration after capping with the SPION^52^, the broad band OH stretch, and H-bonding, which may be caused by the presence of the phenolic compound^53,65,66^. The SPIONs synthesis without oxidation to other iron oxides is confirmed by a noticeable band at 585 cm^−1^, attributed to Fe–O stretching^56^ and 1636 cm^−1^ corresponds to the stretching vibration of the N–H bend the amide 1 group. The shifted peak demonstrated the protein functionality in the SPIONs formation that serves as a minimum agent and capping agent^67^. Additionally, the capping agent provides surface stabilization of SPIONs to avoid agglomeration. TEM explains that SPIONs also feature more solitary, almost spherical NPs and a small aggregation of primary particles^68–70^. It is also possible to see variations in SPION aggregation states from TEM pictures. The particle diameter ranges (20–24 nm) display a modest size distribution. These numbers closely match crystallite size predictions made using XRD profile parameters^52^.

The antimicrobial activity of SPIONs demonstrated that SPIONs minimize the microbial growth of the tested strains with an increase in the activity as the concentration of the SPIONs increased. Eventually, the investigation found that SPIONs inhibited both Gram +ve and Gram −ve bacteria, as well as *Candida albicans* isolate, and gram-positive bacteria being more affected as reported by^67,71^. The MIC of synthesized SPIONs was determined and the results demonstrated the bactericidal effect of SPIONs against all tested isolates. Furthermore, the most affected isolate was *Staphylococcus aureus* with MIC of 3 μg/mL. So, the present investigation displayed the role of the SPIONs to act upon the drug-resistant pathogens that the biocompatible SPIONs exhibit a high specific surface area as well as the small size that enable them to penetrate the membrane of the bacterial cells of and disrupt membrane permeability^72^. Moreover, SPIONs may generate oxidative stress via reactive oxygen species leading to membrane permeability disruption^49,73,74^.

The antioxidant activity results demonstrated the strong antioxidant properties of the SPIONs. The impressive antioxidant qualities of NPs have been shown in earlier investigations^75,76^. The SPIONs either give DPPH radicals electrons or exchange hydrogen atoms with them to neutralize free radicals. Thus, the oxidation process can be prevented, protecting proteins, nucleic acids, carbohydrates, and lipids from oxidative damage^77^. Due to their excellent antioxidant activity, greenly biosynthesized SPIONs can, therefore, be promising candidates as useful natural antioxidants for health preservation against different oxidative stress associated with degenerative diseases. The potency of anti-inflammatory compound was tested, and the results revealed that the SPIONs were efficient against protein denaturation.

Dye degradation is one of the useful uses for the SPIONs. The cationic methylene blue (MB) dye is one of the water contaminants that depletes the dissolved oxygen in water and endangers the aquatic system. The catalytic efficiency of SPIONs was evaluated by using reduction of the MB dye in the presence of NaBH_4_ at room temperature via the electron transfer of BH_4_^-^ ions. The results showed that addition of the SPIONs can produce fast reduction of the MB (50%) of the initial concentration after 4 h in the presence of NaBH_4_. This indicates that the green biosynthesized SPIONs were effective biocatalysts as they degraded the methylene blue dye^52,78^.

Magnetic-hyperthermia: The SPIONs have a high M_*S*_ and a tiny particle size of 20 nm for the efficient HT application methodology; this excess in heating is the basis for magnetic induction heating applications in cancer cell therapy, as reported in^52,79^. In a short time, they were higher than^52,79,80^ depending on the concentration, which indicates the high M_*S*_ of SPIONs.

In conclusion, a sustainable green technique was employed to synthesize SPIONs utilizing *Citrus sinensis* peels extract as stabilizing, minimizing and capping agent. The iron nanoparticles have been characterized by FT-IR, XRD and UV–VIS spectroscopy. SPIONs are a practical therapeutic choice for treating various infections because they demonstrated effective antimicrobial, antioxidant, anti-inflammatory activities in compare with standard drug. This investigation also displayed an excellent catalytic performance of SPIONs during decreased of methylene blue dye which can accumulate in wastewater and affect aquatic life, human health, and ecosystem adversely. The discovered characteristics and HT of SPIONs may explain their applicability in future varied biological applications, such as cancer cell treatment monitoring combined with HT, as Fe_3_O_4_ SPIONs have better SAR and biocompatibility. More in vivo researches and clinical trials are required even though these medications appear to be promising.

### Supplementary Information

Supplementary Tables.

## Data Availability

All data are presented in this published article and supplementary file.
