# Supplementary material for: Green synthesis of biocompatible Fe3O4 magnetic nanoparticles using Citrus Sinensis peels extract for their biological activities and magnetic-hyperthermia applications
Source: Sci Rep. 2023 Nov 3;13:19000. doi: 10.1038/s41598-023-46287-6 (PMC10624884; doi:10.1038/s41598-023-46287-6)
Supplement: Supplementary file 1 — Supplementary Tables. [file 41598_2023_46287_MOESM1_ESM.docx]

Table S1: Antimicrobial activity and MIC of Fe_3_O_4_ SPIONs against various multi-drug resistant isolates

| Diameter of inhibition zones (mm) | | | | | | |
| --- | --- | --- | --- | --- | --- | --- |
| Concentration (μg/ml) | **S. aureus** | **St. mutans** | **B. subtilis** | **E. coli** | **K. pneumonia** | **C. albicans** |
| 50 | 19.8 | 30.5 | 20.2 | 21.4 | 11 | 11.2 |
| 100 | 27.3 | 34.2 | 21.5 | 23.6 | 12.5 | 14.3 |
| 200 | 28.2 | 36 | 21.9 | 25.8 | 12.9 | 15 |
| 300 | 28.2 | 37 | 22.4 | 27.2 | 13.5 | 17.4 |
| 400 | 30 | 37.3 | 22.3 | 30 | 14.7 | 22.6 |
| (MIC) μg/ml | 3 | 6.5 | 6.5 | 12.5 | 50 | 25 |

Table S2. Reported literature on SPIONs and SAR values.

| Samples | Nanocomposite | Concentration  mg/ml | VSM emu g^−1^ | SAR  (W/g) | Particle size (nm) | Frequency  (kHz) | References |
| --- | --- | --- | --- | --- | --- | --- | --- |
| Fe_3_O_4_ | Magnetic GO–lignin | 0.5 | 17.2 | 121 | 70 | 100-400 | 13 |
| Fe_3_O_4_ | PEG | 0.2g | 21 | 1.4 | 33 | 142 | 53 |
| Hydrogel-Fe_3_O_4_ | Graphene oxide | 1 | 75 | 67.7 | ------- | 400 | 54 |
| Fe_3_O_4_ | Dendrimer | 1.12-1.85 | 40-57 | 179 | 7.3 | 87-340 | 55 |
| Fe_3_O_4_ | ---------- | 10 | 102 | 286 | 22 | 290 | Our work |
